# Robust Cell Size Checkpoint from Spatiotemporal Positive Feedback Loop in Fission Yeast

**DOI:** 10.1155/2013/910941

**Published:** 2013-07-11

**Authors:** Jie Yan, Xin Ni, Ling Yang

**Affiliations:** ^1^School of Mathematical Sciences, Soochow University, Suzhou 215006, China; ^2^Center for Systems Biology, Soochow University, Suzhou 215006, China

## Abstract

Cells must maintain appropriate cell size during proliferation. Size control may be regulated by a size checkpoint that couples cell size to cell division. Biological experimental data suggests that the cell size is coupled to the cell cycle in two ways: the rates of protein synthesis and the cell polarity protein kinase Pom1 provide spatial information that is used to regulate mitosis inhibitor Wee1. Here a mathematical model involving these spatiotemporal regulations was developed and used to explore the mechanisms underlying the size checkpoint in fission yeast. Bifurcation analysis shows that when the spatiotemporal regulation is coupled to the positive feedback loops (active Cdc2 promotes its activator, Cdc25, and suppress its inhibitor, Wee1), the mitosis-promoting factor (MPF) exhibits a bistable steady-state relationship with the cell size. The switch-like response from the positive feedback loops naturally generates the cell size checkpoint. Further analysis indicated that the spatial regulation provided by Pom1 enhances the robustness of the size checkpoint in fission yeast. This was consistent with experimental data.

## 1. Introduction

In order to maintain proper size, dividing cells need to time mitosis carefully. Previous analyses performed in fission yeast suggested that there is a homeostatic mechanism that can maintain the appropriate cell size [1–3]. The cell is allowed to enter mitosis only after it reaches a critical size (size checkpoint). Experimental data also showed that cells smaller than critical size had to grow until they reached the threshold value. This period is called the size-dependent phase, or sizer. Then, after a fixed period, called as timer, the cells completed mitosis. Daughter cells that are larger than critical size when produced can undergo mitosis without going through the sizer phase [3]. Some higher eukaryotes such as *Xenopus laevis* [4, 5], Drosophila [6], animal cells [7], and HeLa cells [8] also have similar methods of size control.

Biological experimental data indicate that the rate of cyclin protein synthesis may increase as the cell grows [9]. This may be one mechanism underlying size control. Previous mathematical models have explored the nonlinear dynamic properties of the temporal regulation of cell cycle events [10–12]. The cyclin protein synthesis rate is assumed to increase as the cell grows, and it exhibits a bistable relationship with MPF. This bistability, which is generated by the positive feedback loops in the cell cycle, is responsible for the mitosis initialization [9, 13]. In this way, cell size is linked to entry into mitosis.

Recent evidence has shown that the cell polarity protein kinase Pom1 forms a polar gradient from the ends of the cylindrical cell to its center [14, 15]. In this way, it can provide spatial information that can be used to regulate the mitosis inhibitor Wee1. This spatial regulation links cell size directly to mitosis, and it may play a critical role in size control.

In summary, cell size is coupled to the progression of the cell cycle through the rates of synthesis of cyclin proteins and the direct spatial information provided by Pom1. The results of the present study show that when spatial regulation and the rate of synthesis rate are both coupled to temporal positive feedback loops, a bistable response generates the cell size checkpoint. Bifurcation analysis shows that the concentration of MPF can exhibit a bistable steady-state relationship with the rate of synthesis of cyclin proteins or the concentration of Cdr (downstream of Pom1) alone. The size checkpoint is naturally built into the system in the form of dual regulations of the rate of synthesis and the Pom1 gradient. Stochastic analysis then showed that the direct spatial regulation can allow temporal positive feedback to enhance the robustness of the cell size checkpoint in fission yeast, which is consistent with the experimental data. 

## 2. Results and Discussion

### 2.1. Mathematical Modeling

**Figure 1:**
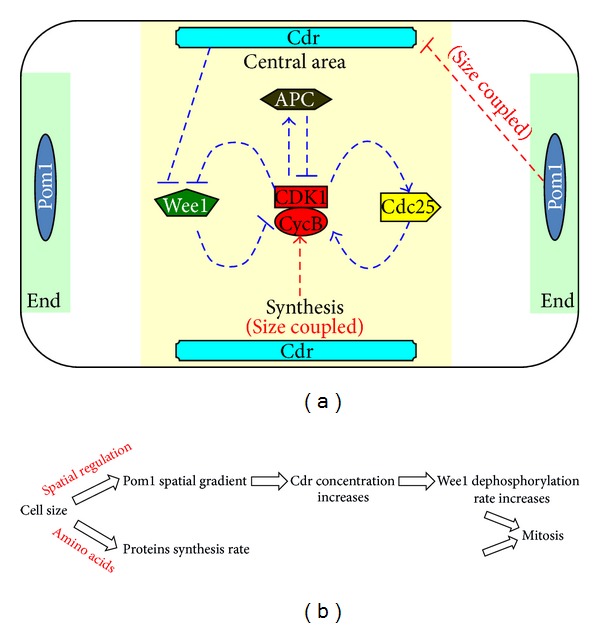
(a) Regulatory network of the cell cycle in fission yeast. (b) Two ways in which cell growth is coupled to cell division: the rate of synthesis of Cdc13 and the direct spatial regulation provided by Pom1.

The upper panel of Figure 1(a) shows a schematic diagram of the protein interaction in G2-M phase. The regulatory network includes a negative feedback loop: active Cdc2/Cdc13 dimer (MPF) inhibits itself by promoting the production of APC complexes and thus promotes cyclin ubiquitination and degradation. In addition to this negative feedback loop, regulation during the G2/M phase also involves two positive feedback loops: there is a phosphorylatable tyrosine residue (the Tyr-15 residue) at the active site of Cdc2. If the active site is phosphorylated, MPF is inactive. Wee1, a kind of tyrosine kinases, can inactivate Cdc2/Cdc13 (MPF) in this way. MPF can also phosphorylate Wee1 to repress its activity. On the other side, tyrosine phosphatases Cdc25 can remove the inhibitory phosphate group on Tyr-15 to activate MPF. In return, MPF can promote the activity of Cdc25 by increasing the phosphorylation rate of Cdc25. In summary, active Cdc2/Cdc13 activates its activator Cdc25 and inactivates its inhibitor Wee1. During the cell cycle, Cdc13 is continuously synthesized from amino acids. The rate of synthesis of Cdc13 increases as the cell grows (Figure 1(b)).

Besides, Cdr proteins also couple cell growth to cell division through a size sensing mechanism involving Pom1. Several previous works have identified the function of the Pom1 pathway [14–16]. The cell polarity protein kinase Pom1 is a cell polarity protein kinase, which can form a spatial gradient that is greatest at the ends of the cylindrical cell and least in the middle of the cell. Cdr which locates near the center of the cell can suppress the activity of Wee1 and so promote mitosis. Pom1 phosphorylates Cdr to inhibit its activity. The size-dependent relief of this inhibition can repress Wee1 to promote the initialization of mitosis.

The network was then transferred into a set of ordinary differential equations using the principles of biochemical kinetics. The initial size of a WT daughter cell was normalized to 1. The model was adapted from the models constructed by James Ferrell's group [17] and Novak-Tyson's group [11]. However, different from their models, we also took the spatial information provided by Pom1 into consideration.

A detailed mathematical model is presented in Section 4.

### 2.2. Bifurcation Analysis

Experimental observations have provided some evidences of size checkpoint [2]. If the initial size of a *cdc2-33* fission yeast cell was smaller than 12 *μ*m, a marked negative relationship was observed between the extension length and the initial size. However, the extension length was not found to be significantly related to cell size at initial sizes larger than 12 *μ*m. This critical size that determined whether the cell could begin mitosis was the size checkpoint. Besides, Rupeš and colleagues also showed that cells smaller than critical size had to grow until they reached the threshold value. If the birth size of the fission yeast is larger than the critical size, the cell can undergo mitosis without additional time delay [3]. This critical size also indicates the existence of the size checkpoint. 

Earlier experimental studies have revealed that the steady state of MPF shows a hysteretic steady-state response relationship with the concentration of cyclin B [13]. Mathematical models have established that the concentration of MPF has a bistable relationship with the rate of synthesis of the cyclin proteins. This bistability is attributable to the positive feedback loops (active Cdc2 promotes its activator Cdc25 and suppresses its inhibitor Wee1) [18, 19]. In our model, the positive feedback loops rely both on the rate of synthesis and on the spatial regulation involving Wee1. In this way, the coupling between cell size and cell division is more realistic in this model.

**Figure 2:**
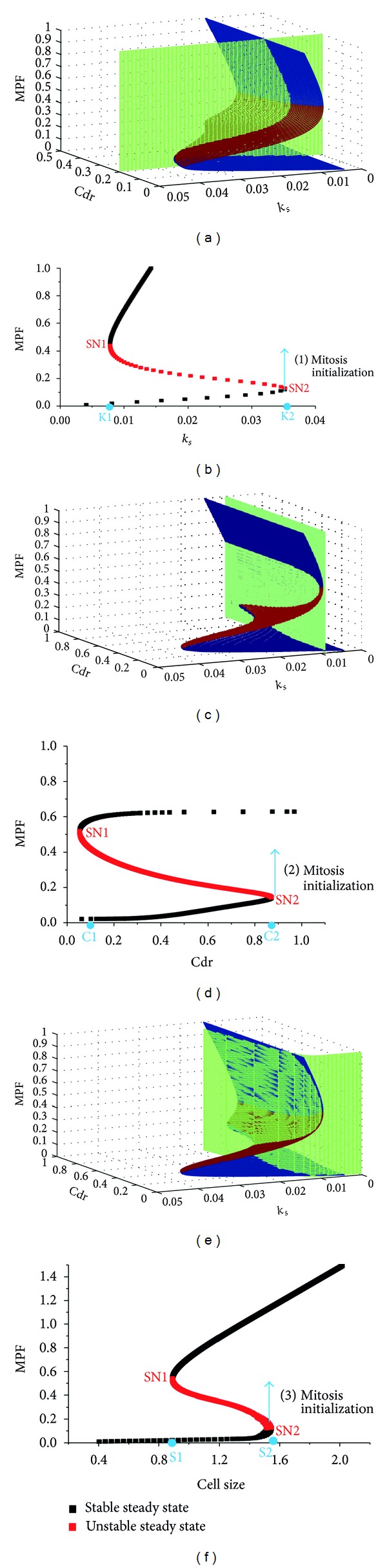
(a)(c)(e) The steady state of MPF with different given rates of synthesis (*k*_*s*_) and different given concentrations of Cdr (cdr). The dark blue areas indicate the stable steady state of MPF, and the dark red areas indicate the unstable steady state of MPF. (a) The green vertical plane represents the variation in the rates of Cdc13 synthesis as the cell grows for a fixed concentration of Cdr. The plane intersects with the bent surface, forming an S-shaped curve. (b) When this curve is displayed in two-dimensional space, it represents the relationship between MPF and the rate of synthesis. (c) The green vertical plane represents the variation in the concentration of Cdr as the cell grows for a fixed rate of Cdc13 synthesis. The plane intersects with the bent surface, forming an S-shaped curve. (d) When this curve is displayed in two-dimensional space, it represents the relationship between MPF and the concentration of Cdr. (e) The vertical green surface represents the relationship between the concentration of Cdr and the rate of synthesis of Cdc13 as the cell grows. The vertical green surface intersects with the bent surface, forming an S-shaped curve. (f) When this curve is displayed in two-dimensional space, it represents the relationship between MPF and cell size.

To demonstrate how the rate of synthesis of Cdc13 and the concentration of Cdr both affect the activation of MPF, we first calculated the steady state of MPF for a given rate of Cdc13 synthesis and a given concentration of Cdr, when Cdc13 was made nondegradable (during the period prior to mitosis, the concentration of APC remains at a low and constant level) [20]. The results are presented in three-dimensional space (the green surfaces in Figures 2(a), 2(c), and 2(e)).

If the regulation related to the rate of synthesis is solely considered, then the vertical plane Cdr = 0.2 (which represents a fixed concentration of Cdr, which occurs when Pom1 spatial regulation is blocked) intersects with the surface at an S-shaped curve (Figure 2(a)). The bifurcation analysis shows that the steady state of MPF has a bistable relationship with the rate of synthesis (Figure 2(b)). As the cell grows, the rate of synthesis of Cdc13 increases and the concentration of MPF accumulates in turn. When the rate of synthesis passes point K2 in Figure 2(b) as the cell grows, the low stable branch disappears and the MPF has to jump to the upper stable branch (arrow (1)). And the mitosis begins. In this way, the rate of synthesis of Cdc13 contributes to the function of the size checkpoint.

Similarly, if the regulation related to the Pom1 pathway is solely considered, the vertical plane synthesis rate = 0.009 (which represents a fixed synthesis rate of Cdc13) intersects with the bent surface along an S-shaped line (Figure 2(c)). The bifurcation analysis shows that the steady state of MPF also has a bistable relationship with Cdr (the downstream of Pom1) when the direct spatial regulation is linked to the positive feedback loops (Figure 2(d)). As the cell grows, the concentration of Cdr increases (due to a reduction in regulation provided by Pom1). Then the concentration of MPF also accumulates along the lower branch. When the concentration of Cdr passes point C2 as the cell grows (Figure 2(d)), the low stable branch disappears and MPF has to jump to the upper stable branch (arrow (2)). Then mitosis begins. In this way, the direct spatial regulation provided by the Pom1 pathway also contributes to the function of the size checkpoint.

In the real-world cell cycle, these two previous regulations both contribute to the coupling of the cell size and cell division. The steady state of MPF in real-world systems was assessed as follows. First, the relationship between the rate of synthesis of Cdc13 and the concentration of Cdr as the cell grows was calculated. Then the vertical surface, which represents the variation in the rate of synthesis rate and the concentration of Cdr as the cell grows, was intersected with the steady state surface (Figure 2(e)). As in the sole regulation scenarios, the line of intersection is S-shaped. This means that the steady state of MPF continues to exhibit bistability with cell size when the spatial regulation and the rate of synthesis are both involved in the positive feedback loops. After that we directly linked the steady state of MPF to the cell size through bifurcation analysis (Figure 2(f)). Figure 2(f) shows that the concentration of MPF increases as the cell grows. When the cell size reaches the threshold, about 1.5 (size checkpoint, S2 in Figure 2(f)), MPF switches to the upper branch (arrow (3)). Then the cell undergoes mitosis.

Then we further summarized the relationship between the bifurcation analysis and the size checkpoint. The bifurcation analysis shows that MPF exhibits the bistability with the cell size. There is a critical cell size S2 (corresponding to saddle node point SN2): if the cell size is smaller than S2, MPF stays in low level; if the cell size passes S2 point, the low stable branch disappears and MPF has to jump to the upper stable branch. As we mentioned previously, experimental studies [1, 2, 21, 22] have shown that a cell will not begin mitosis until it grows to a critical size. Therefore, this saddle node point SN2 naturally performs the role of a check point: before the size reaches S2, cell remains in G2 state (low MPF); once the cell passes S2 point, MPF can jump to the upper branch to trigger mitosis.

**Figure 3:**
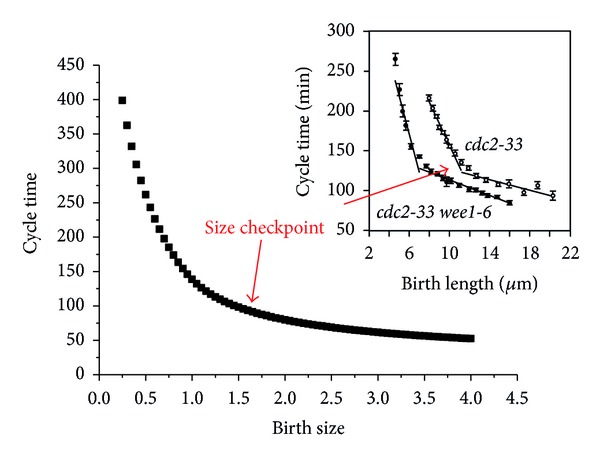
The numerical simulation was used to confirm the size checkpoint. The *x*-axis represents the initial size of the fission yeast in the model. The *y*-axis represents the points in the cell cycle when the birth size changes from 0.25 to 4. The red arrow indicates the size checkpoint. A previous experimental data [2] is inserted to compare with the simulation.

After that, a numerical simulation was used to check the size checkpoint (Figure 3). The initial size of the model varied from 0.25 to 4. The result shows that when the initial cell size is smaller than 1.5, the cycle time shows a significantly negative relationship with the initial size. However, if the initial size exceeded 1.5, then the cycle time was mostly independent of the initial cell size. This result accords with the previous experimental data in yeast (the inserted figure in Figure 3 [2]). In this way, 1.5 is established as the size checkpoint. The result of the simulation is also consistent with the bifurcation analysis shown in Figure 2(f), where the critical size for the mitosis initialization is about 1.5.

In summary, the concentration of MPF exhibits a bistable steady-state relationship with cell size, which depends on the spatiotemporal positive feedbacks. This bistability naturally produces the size checkpoint.

### 2.3. Stochastic Analysis

Experimental evidence showed that some intrinsic stochastic noise (such as random cell production and collisions between molecules) and extrinsic stochastic noise (such as variations in the environment) will result in fluctuations in gene expression [23]. In this way, processes related to the cell cycle may vary from cell to cell within a population, over time, and even within a single cell. The present study not only coupled cell size to the rate of synthesis of Cdc13 but also to the direct spatial regulation provided by the Pom1 pathway. This direct spatial regulation may help the size checkpoint resist interference from different sources and keep cell size coupled to cell division. 

To evaluate the impact of random fluctuation on the cell cycle, some stochastic noise was introduced to the present model: (1) each parameter in the deterministic model was multiplied by a stochastic factor, which was randomly chosen from the normal distribution with *μ* = 1 and *σ* = 0.016 (*μ* represents the mean value and *σ* represents the variance of the distribution). In this way, the cell cycle can fluctuate near the deterministic value. (2) After mitosis, the cell did not divide into two identical daughter cells. Asymmetrical division was characterized by a normal distribution with *μ* = 0.5 and *σ* = 0.016.

**Figure 4:**
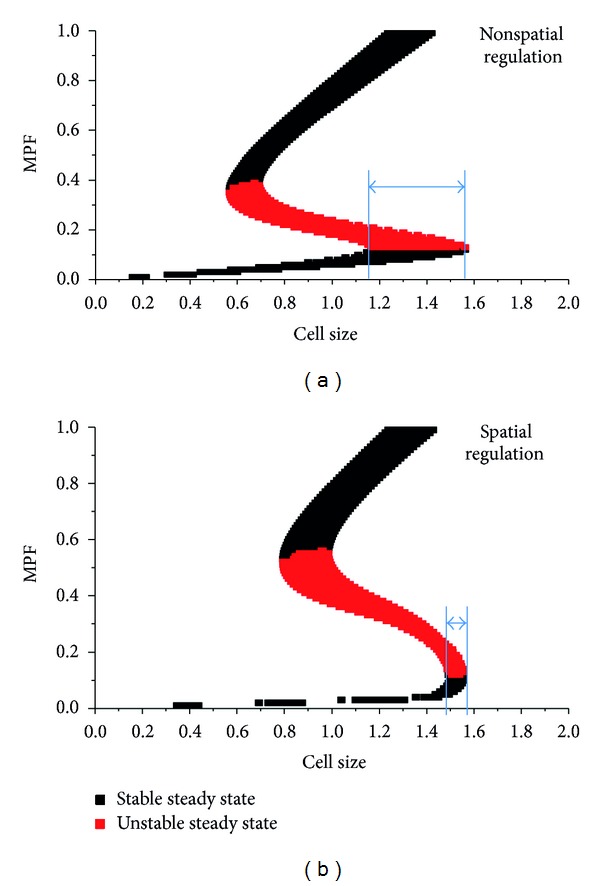
The relationship between the steady state of MPF and cell size in (a) a system without spatial regulation and (b) a system with spatial regulation. The red points represent the unstable steady state of MPF, and the black points represent the stable steady state of MPF. The blue bar indicates the range of the size checkpoint.

Then the model was used to determine if the spatial regulation can help the size checkpoint resist the fluctuations of the system. When the stochastic factor was disturbed, the size check point was calculated 100 times with and without the Pom1 spatial regulation. Results are shown in Figure 4.

Figure 4(a) shows that if there is no spatial regulation in the system (i.e., if cell size is linked to cell division only through the rate of synthesis and if the concentration of Cdr is fixed at 0.5), the size checkpoint varies from 1.15 to 1.55 in the presence of stochastic noise. However, if spatial regulation is taking place in the system, the size checkpoint changes from 1.48 to 1.56, which is much narrower than the one shown in Figure 4(a). In this way, even with the interference produced by stochastic noise, the cell must still exceed a strict size checkpoint. The comparison indicates that the direct spatial regulation provided by the Pom1 pathway can ensure tight coupling between cell size and the cell division.

**Figure 5:**
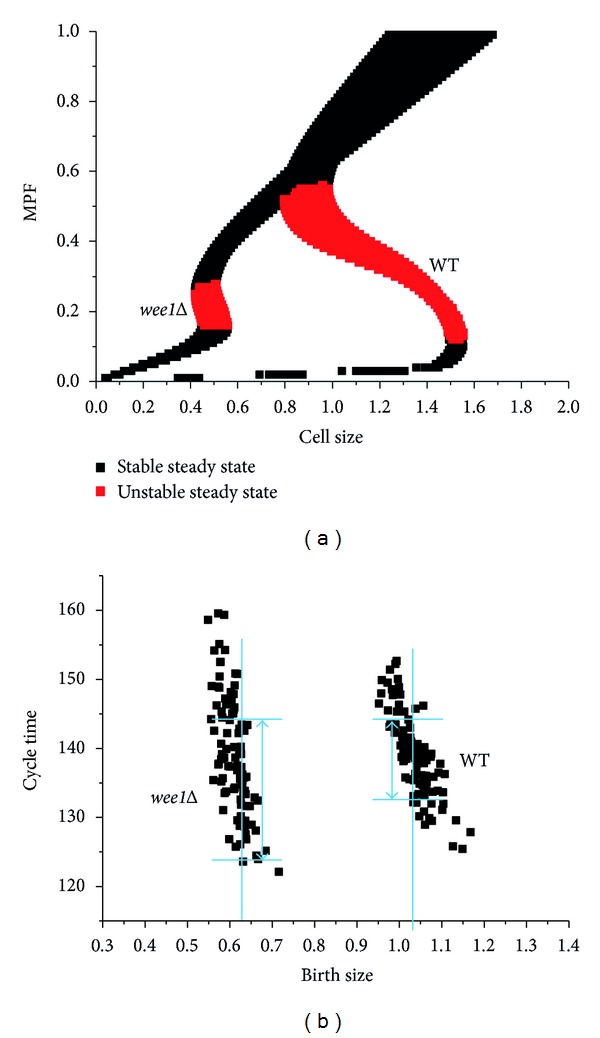
(a) Bifurcation analysis in *wee1Δ* and in WT fission yeast. The red points represent the unstable steady state of MPF, and the black points represent the stable steady state of MPF. The blue bar indicates the range of the size checkpoint. (b) Stochastic numerical simulation in *wee1Δ* and WT. The *x*-axis and *y*-axis represent the initial size and the duration of the cell cycle of the fission yeast. The blue bars represent the range of the cycle time for a given birth size in *wee1Δ* and in WT fission yeast.

Because direct spatial regulation works through the mitosis inhibitor Wee1, *wee1Δ* may show a weak ability to resist interference. This mathematical model was used to assess the size checkpoint in *wee1Δ* (*k*5 = 0.15, decreases from 2 in WT, the stochastic noise presents as mentioned previously). Bifurcation analysis shows the size at which cells undergo mitosis in *wee1Δ* to be about half of that in WT (Figure 5(a)). This result accords with the previous experimental data that the *wee1Δ* cell divided at a half size of WT [2]. However, in the presence of random disturbances, the width of the size checkpoint in *wee1Δ* was found to be twice of that of WT. These theoretical results are consistent with observations made in earlier experiments [2]. Experimental observation showed that *wee1Δ* fission yeast exhibited larger variance in the duration of the cell cycle for any given initial size (Figure 1(b) of a previous study) [2]. Because the duration of the cell cycle includes the time required for the cell to reach the size checkpoint (sizer phase) and the fixed time, which is independent of other factors (timer phase) [24]. This indicates that the variation of the size checkpoint is larger in *wee1Δ* than in WT. Furthermore, Table 1 in [2] summarized that the variation in the division length was about twice as large in *wee1Δ* as in WT, and the variation in cycle time was increased in the similar way. Therefore, it means that the size control in *wee1Δ* is not as strict as that in WT. In this way, the size checkpoint in *wee1Δ* is less robust than in WT.

Then the numerical simulation results of *wee1Δ* and WT fission yeast were compared to those produced in earlier experiments. During the simulation, stochastic factors continued to act on the cell. And the initial concentrations of proteins and initial size were given in the deterministic model. After every division, the system listed the initial cell size and initial concentrations of relevant proteins for the next cell cycle. The results of the simulation are shown in Figure 5(b): the overall range of the duration of the cell cycle was similar in *wee1Δ* and WT. However, for a given initial size, the range of the duration of the cell cycle was always larger in *wee1Δ* than in WT. The large variation in the length of the cycle time can be attributed to weakness in the size checkpoint control. These results are consistent with those of a previous experiment published by Novak and Tyson [2]. 

In conclusion, the direct spatial regulation provided by Pom1 can enhance the robustness of the size checkpoint and couple cell size to cell division.

## 3. Discussion

Although a group of models have investigated the temporal regulation of cell cycle [10, 25–27], most of them did not consider the direct spatial regulation provided by Pom1. In our model, we take this regulation into account. Vilela and colleagues built a mathematical model incorporating the Pom1 pathway [16]. However, they paid more attention on the formation of the Pom1 gradient and overpassed the link between bistability and size checkpoint. In our model, we specified that the critical size S2 (corresponding to saddle node of the lower branch SN2) is the cell size checkpoint and focused on the robustness of the size checkpoint.

Since the function of the Pom1 pathway has not been understood until 2009, our previous work related the size checkpoint to the cytoplasmic-to-nuclear size ratio. In the present work, we revealed that the underlying mechanism of size checkpoint is the saddle node bifurcation.

Bifurcation analysis of *wee1Δ* (Figure 5(a)) showed that although the range of the cell size checkpoint is larger than that of WT, it is still narrower than that of systems without spatial regulation (Figure 4(a)). This is because the spatial regulation is still assumed to work in *wee1Δ* (k5 remains 7.5% of WT, not 0 in *wee1Δ*). Even this weak spatial regulation can enhance the robustness of size checkpoint significantly. Therefore, the direct spatial regulation provided by Pom1 is thought to play a more important role in coupling cell growth to cell division.

Cell size checkpoints are present in many kinds of cells. A robust cell size checkpoint is required for the maintenance of appropriate cell size during proliferation. Although only spatial regulation was reflected in the present model of fission yeast, other cells, such as frog eggs, may also have similar ways of transferring spatial information directly, but this has not been experimentally established. Unlike that of fission yeast, the spatial regulation of oocytes takes place in a spherical space [12].

In the present study, a mathematical model was used to investigate the manner in which cell size can be coupled to the cell division in fission yeast. As the cells grow, the rate of synthesis of Cdc13 increases. However, the relief gradient offered by Pom1 can reduce the concentration of Cdr, which reduces the ability of Cdr to inhibit Wee1. The novel dynamics shown in the present model can be used to evaluate the direct spatial regulation provided by Pom1 and to examine its impact on cell checkpoints. The positive feedback loops were found to depend on spatial regulation and to generate a switch-like MPF response, which naturally produces the endogenetic size checkpoint. This direct spatial relation was found to protect the size checkpoint from fluctuations in gene expression.

## 4. Methods and Materials

The mathematical models of the cell cycle have been extensively studied. We adapted the parameters from the classic models of Ferrell's and Tyson's group. Besides, we also added the effect of the Pom1 pathway on Wee1 regulation. In other words, we introduced the spatial regulation to the system. The ordinary differential equations for this mathematical model are as follows:

(1) $$\begin{array}{c} V=V_{0}*e^{\mu t} \end{array}$$

(2) dCdc13(t)dt=k1∗V−k3∗(Cdc2tot−Cc(t)−Ccp(t)            − Cptp(t)−MPF(t)) ∗Cdc13(t)+k4∗CC(t)−k2 ∗(Apc(t)+Apc_basal)∗Cdc13(t),

(3) dCc(t)dt=k3∗(Cdc2tot−Cc(t)−Ccp(t)       − Cptp(t)−MPF(t))∗Cdc13(t) −k4∗CC(t)+k8∗Ccp(t)∗Cdc25(t) +k9∗Ccp(t)∗(k10−Cdc25(t)) −k5∗Wee1(t)Cc(t)−k6 ∗(k7−Wee1(t))∗Cc(t),

(4) dCcp(t)dt=k5∗Wee1(t)Cc(t)+k6 ∗(k7−Wee1(t))∗Cc(t)−k8 ∗Ccp(t)∗Cdc25(t)−k9∗Ccp(t) ∗(k10−Cdc25(t))+k12∗y4−k11 ∗y3−k2∗(y9+Apc_basal)∗y3,

(5) dCcpt(t)dt=k11∗Ccp(t)−k12∗Ccpt(t) −k8∗Cdc25(t)∗Ccptp−k9 ∗(k10−Cdc25(t))∗Ccptp(t) +k5∗Wee1(t)∗MPF(t)+k6 ∗(k7−Wee1(t))∗y5−k2 ∗(Apc(t)+Apc_basal)∗Ccptp(t),

(6) dMPF(t)dt=k8∗Cdc25(t)∗Ccpt(t) +k9∗(k10−Cdc25(t))∗Ccpt(t) −k5∗Wee1(t)∗MPF(t)−k6 ∗(k7−Wee1(t))∗MPF(t) −k2∗(APC(t)+Apc_basal)∗MPF,

(7) dCdc25(t)dt=k13∗MPF(t)k14k27k14+MPF(t)k14 ∗(k10−Cdc25(t))−k15∗Cdc25(t),

(8) $$\begin{array}{c} \mathrm{rel}=V*7, \end{array}$$

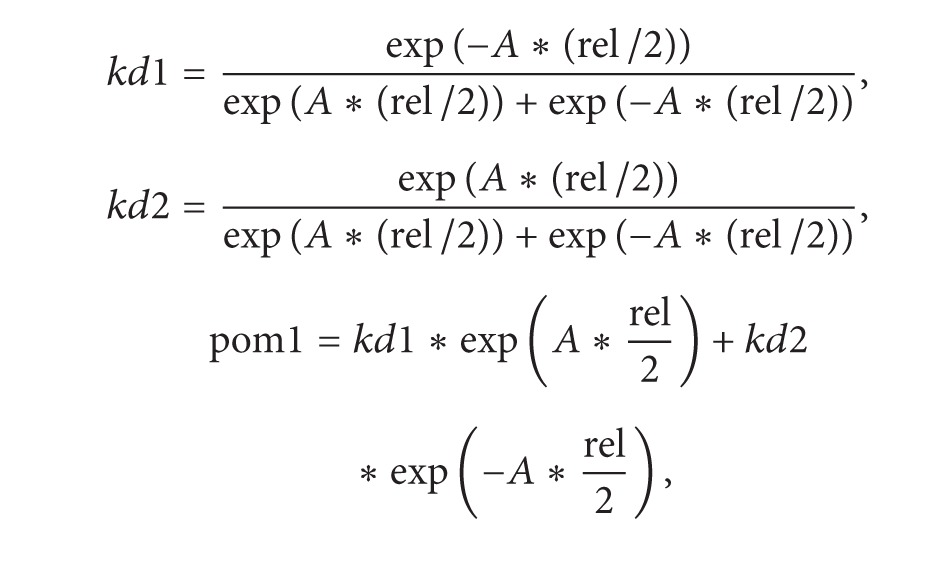
(9)

(10) $$\begin{array}{c} \text{Cdr}=\frac{k\text{cdr}\;\mathrm{on}}{k\text{cdr}\;\mathrm{on}+k\text{cdr}\;\mathrm{off}*\text{pom}1^{n}/\left(\text{pom}1^{n}+K^{n}\right)\;} \end{array}$$

(11) dWee1(t)dt=−k16∗(MPF(t)+cdr)k17k28k17+(MPF(t)+cdr)k17 ∗Wee1(t)+k18∗(k7−Wee1(t)),

(12) dPlo1(t)dt=k19∗MPF(t)k20k30k20+MPF(t)k20 ∗(k21−Plo1(t))−k22∗Plo1(t),

(13) dApc(t)dt=k23∗Plo1(t)k24k30k24+Plo1(t)k24∗(k25−Apc(t)) −k26∗Apc(t).

In our model, the change rate of Pom1 at location *x* is given by a kinetic equation, where the first term is the rate of diffusion and the second term is its rate of degradation.

(14) ∂Pom1(x,t)∂t=m∗∂2Pom1(x,t)∂x2−k ∗Pom1(x,t).

Here *m* represents the diffusion coefficient of Pom1 in fission yeast. And *k* represents the degradation coefficient of Pom1.

To make the system simpler, we supposed that the gradient of Pom1 can be formed rapidly. Therefore the concentration of Pom1 at *x* is set in quasi-steady state as follows:

(15) $$\begin{array}{c} m*\frac{d^{2}\text{Pom}1\left(x\right)}{dx^{2}}=k*\text{Pom}1\left(x\right),\\ \text{Pom}1\left(x\right)=kd1*e^{x*A}+kd2*e^{-x*A}, \end{array}$$

where $A=\sqrt{k/m}$

(16) $$\begin{array}{c} kd1=\frac{\exp\left(-A*\left(\mathrm{rel}/2\right)\right)}{\exp\left(A*\left(\mathrm{rel}/2\right)\right)+\exp\left(-A*\left(\mathrm{rel}/2\right)\right)},\\ kd2=\frac{\exp\left(A*\left(\mathrm{rel}/2\right)\right)}{\exp\left(A*\left(\mathrm{rel}/2\right)\right)+\exp\left(-A*\left(\mathrm{rel}/2\right)\right)}. \end{array}$$

Here *rel*⁡ represents the amplified size of the fission yeast. These parameters are estimated from the model of Vilela and colleagues [16]. It is notable that the birth size of the fission yeast is normalized to 1 in our model. However, the birth size of fission yeast is 7 *μ*m in Vilela's model. Therefore, we amplified the cell size to 7-fold when applying the parameters of Vilela's model:

(17) $$\begin{array}{c} \mathrm{rel}=V*7. \end{array}$$

The activity of Cdr is repressed by the Pom1 at the central zone of the fission yeast. Thus we only need to consider the concentration of Pom1 at the center of the fission yeast:

(18) $$\begin{array}{c} \text{pom}1=kd1*\exp\left(A*\frac{\mathrm{rel}}{2}\right)+kd2*\exp\left(-A*\frac{\mathrm{rel}}{2}\right). \end{array}$$

The change rate of Cdr is formulated as follows:

(19) dCdr(t)dt=−kcdr off⁡∗Pom1nKn+Pom1n∗Cdr(t) +kcdr on⁡∗(1−Cdr(t)).

Similarly, to simplify the model, we assumed that the concentration of Cdr is set in steady state. Thus the ordinary equation can be transformed into an algebraic equation:

(20) $$\begin{array}{c} \text{Cdr}=\frac{k\text{cdr}\;\mathrm{on}}{k\text{cdr}\;\mathrm{on}+k\text{cdr}\;\mathrm{off}*\text{pom}1^{n}/\left(\text{pom}1^{n}+K^{n}\right)}. \end{array}$$

The parameters are as follows:

(21) $$\begin{array}{c} k1=0.01056;\;\;k2=1;\;\;k3=10;\;\;k4=0.1;\\ k5=2;\;\;k6=0.1;\;\;k8=2;\;\;k9=0.05;\\ k7=1;\;\;k10=1;\;\;k11=0.4;\;\;k12=0.002;\\ k13=10;\;\;k14=4;\;\;k15=1;\;\;k16=10;\\ k17=4;\;\;k18=1;\;\;k24=4;\;\;k20=4;\\ k21=1;\;\;k25=1;\;\;k22=0.2;\;\;k23=10;\\ k26=0.2;\;\;k19=10;\;\;k27=0.8;\;\;k28=0.8;\\ k29=1.3;\;\;k30=1.3;\;\;\text{cdk}\;\text{tot}=20;\\ \text{apc}\_\text{basal}=0.01;\;\;k\text{cdr}\;\mathrm{on}=5;\;\;k\text{cdr}\;\mathrm{off}=497;\\ K=0.5;\;\;A=\sqrt{0.12;}\;\;n=9. \end{array}$$

## References

[1] Fantes P, Nurse P (1977). Control of cell size at division in fission yeast by a growth modulated size control over nuclear division. *Experimental Cell Research*.

[2] Sveiczer A, Novak B, Mitchison JM (1996). The size control of fission yeast revisited. *Journal of Cell Science*.

[3] Rupeš I, Webb BA, Mak A, Young PG (2001). G2/M arrest caused by actin disruption is a manifestation of the cell size checkpoint in fission yeast. *Molecular Biology of the Cell*.

[4] Masui Y, Wang P (1998). Cell cycle transition in early embryonic development of *Xenopus laevis*. *Biology of the Cell*.

[5] Wang P, Hayden S, Masui Y (2000). Transition of the blastomere cell cycle from cell size-independent to size-dependent control at the midblastula stage in *Xenopus laevis*. *Journal of Experimental Zoology*.

[6] Edgar BA, Lehner CF (1996). Developmental control of cell cycle regulators: a fly's perspective. *Science*.

[7] Dolznig H, Grebien F, Sauer T, Beug H, Müllner EW (2004). Evidence for a size-sensing mechanism in animal cells. *Nature Cell Biology*.

[8] Takahashi T, Bhide PG, Goto T, Miyama S, Caviness VS (1999). Proliferative behavior of the murine cerebral wall in tissue culture: cell cycle kinetics and checkpoints. *Experimental Neurology*.

[9] Sveiczer A, Tyson JJ, Novak B (2004). Modelling the fission yeast cell cycle. *Briefings in Functional Genomics and Proteomics*.

[10] Sveiczer A, Csikasz-Nagy A, Gyorffy B, Tyson JJ, Novak B (2000). Modeling the fission yeast cell cycle: quantized cycle times in wee1- cdc25Δ mutant cells. *Proceedings of the National Academy of Sciences of the United States of America*.

[11] Novak B, Pataki Z, Ciliberto A, Tyson JJ (2001). Mathematical model of the cell division cycle of fission yeast. *Chaos*.

[12] Qu Z, Weiss JN, MacLellan WR (2004). Coordination of cell growth and cell division: a mathematical modeling study. *Journal of Cell Science*.

[13] Pomerening JR, Sun YK, Ferrell JE (2005). Systems-level dissection of the cell-cycle oscillator: bypassing positive feedback produces damped oscillations. *Cell*.

[14] Martin SG, Berthelot-Grosjean M (2009). Polar gradients of the DYRK-family kinase Pom1 couple cell length with the cell cycle. *Nature*.

[15] Moseley JB, Mayeux A, Paoletti A, Nurse P (2009). A spatial gradient coordinates cell size and mitotic entry in fission yeast. *Nature*.

[16] Vilela M, Morgan JJ, Lindahl PA (2010). Mathematical model of a cell size checkpoint. *PLoS Computational Biology*.

[17] Tsai TY, Yoon SC, Ma W, Pomerening JR, Tang C, Ferrell JE (2008). Robust, tunable biological oscillations from interlinked positive and negative feedback loops. *Science*.

[18] Novak B, Tyson JJ (1993). Numerical analysis of a comprehensive model of M-phase control in *Xenopus oocyte* extracts and intact embryos. *Journal of Cell Science*.

[19] Ferrell JE (2002). Self-perpetuating states in signal transduction: positive feedback, double-negative feedback and bistability. *Current Opinion in Cell Biology*.

[20] Cross FR, Archambault V, Miller M, Klovstad M (2002). Testing a mathematical model of the yeast cell cycle. *Molecular Biology of the Cell*.

[21] Fantes PA (1977). Control of cell size and cycle time in *Schizosaccharomyces pombe*. *Journal of Cell Science*.

[22] Nurse P, Thuriaux P (1977). Controls over the timing of DNA replication during the cell cycle of fission yeast. *Experimental Cell Research*.

[23] Lei J (2009). Stochasticity in single gene expression with both intrinsic noise and fluctuation in kinetic parameters. *Journal of Theoretical Biology*.

[24] Yang L, Han Z, MacLellan WR, Weiss JN, Qu Z (2006). Linking cell division to cell growth in a spatiotemporal model of the cell cycle. *Journal of Theoretical Biology*.

[25] Novak B, Csikasz-Nagy A, Gyorffy B, Chen K, Tyson JJ (1998). Mathematical model of the fission yeast cell cycle with checkpoint controls at the G1/S, G2/M and metaphase/anaphase transitions. *Biophysical Chemistry*.

[26] Sveiczer A, Tyson JJ, Novak B (2001). A stochastic, molecular model of the fission yeast cell cycle: role of the nucleocytoplasmic ratio in cycle time regulation. *Biophysical Chemistry*.

[27] Qu Z, MacLellan WR, Weiss JN (2003). Dynamics of the cell cycle: checkpoints, sizers, and timers. *Biophysical Journal*.

